# Seasonal nearshore ocean acidification and deoxygenation in the Southern California Bight

**DOI:** 10.1038/s41598-022-21831-y

**Published:** 2022-10-26

**Authors:** Samuel A. H. Kekuewa, Travis A. Courtney, Tyler Cyronak, Andreas J. Andersson

**Affiliations:** 1 Scripps Institution of Oceanography, University of California San Diego, La Jolla, CA USA; 2 Department of Marine Sciences, University of Puerto Rico Mayagüez, Mayagüez, PR USA; 3 Department of Marine and Environmental Sciences, Halmos College of Natural Sciences and Oceanography, Nova Southeastern University, Dania Beach, FL USA

**Keywords:** Marine chemistry, Environmental sciences

## Abstract

The California Current System experiences seasonal ocean acidification and hypoxia (OAH) owing to wind-driven upwelling, but little is known about the intensity, frequency, and depth distribution of OAH in the shallow nearshore environment. Here we present observations of OAH and dissolved inorganic carbon and nutrient parameters based on monthly transects from March 2017 to September 2018 extending from the surf zone to the ~ 40 m depth contour in La Jolla, California. Biologically concerning OAH conditions were observed at depths as shallow as 10 m and as close as 700 m to the shoreline. Below 20 m depth, 8% of observations were undersaturated with respect to aragonite, 28% of observations had a pH_T_ less than 7.85, and 19% of observations were below the sublethal oxygen threshold of 157 µmol kg^−1^. These observations raise important questions about the impacts of OAH on coastal organisms and ecosystems and how future intensified upwelling may exacerbate these conditions.

## Introduction

As a result of anthropogenic CO_2_ emissions, ocean warming, acidification, and deoxygenation are expected to intensify globally^1,2^. These changes have raised serious concerns for how marine organisms and communities will be affected, with potential negative impacts expected for many marine ecosystems^1,3,4,5,6^. Quantifying and projecting the trajectories of ocean acidification and hypoxia (OAH) are critical for understanding how ecosystems will be impacted^7^. However, even though predictions of future mean global ocean conditions are fairly robust, it is not fully clear how OAH will interact with local variability across oceanic habitats, ecosystems, and regions, and especially in coastal regions where biological metabolism, inputs from rivers and runoffs, and upwelling may have large impacts^8^.

The west coast of the United States and other eastern boundary upwelling systems (e.g., California, Humboldt, Canary, and Benguela Current Systems) may be particularly vulnerable to OAH due to seasonal wind-driven upwelling that brings deep seawater low in pH and dissolved oxygen (DO) to shallow coastal environments^9–16^. In general, prevailing equatorward winds along the coast in spring and summer drive offshore transport of surface water (i.e., Ekman transport), which is balanced by vertical transport of deep seawater^17^. This seawater has naturally high concentrations of dissolved inorganic carbon (DIC) and nutrients and low levels of oxygen, pH, and aragonite saturation state (Ω_Ar_) owing to metabolic processes that occur below the photic zone^15,18,19^. In addition, these waters carry a gradually increasing anthropogenic carbon signature, which intensifies the already low pH and Ω_Ar_^9,20–22^. Evidence also suggests that upwelling intensity and duration have recently increased because of stronger atmospheric pressure gradients and equatorward winds due to climate change^23–28^.

Ongoing OAH and intensified upwelling have already negatively affected marine organisms on the U.S. west coast, including both natural communities^11,13^ and commercial oyster hatcheries^29^. Future projections suggest that the intensity and duration of exposure to low pH, Ω_Ar_, and DO will increase in this region, which may exceed organisms’ thresholds of pH and DO tolerance, leading to negative effects on ecosystem functioning^5,12,13,25,30–32^. However, to accurately forecast and understand the future effects of OAH in this environment, a detailed characterization of the present-day natural variability of seawater pH and DO at local and regional scales is required (e.g.,^8,12,14^).

Since 1984, the seawater biogeochemistry of the California Current Ecosystem has been monitored quarterly via the California Cooperative Oceanic Fisheries Investigation (CalCOFI) and by the California Current Ecosystem Long-Term Ecological Research programs. However, these large-scale programs do not typically encompass the shallow nearshore environment defined here as the area between the surf zone and the 40 m isobath^33^. This area is particularly important to monitor because many taxa (e.g., mussels, abalone, sea urchins, crustaceans, coralline algae) and ecosystems (e.g., kelp forests, seagrass beds, mussel beds) are highly vulnerable to changes in upwelling dynamics and OAH, and also serve disproportionately important roles for human activities (e.g., aquaculture, tourism, and fisheries). As a result, several research efforts have deployed autonomous sensors in this environment to characterize the high-frequency pH and DO variability in select habitats (e.g.,^12,14,31,34,35^). These efforts have provided critical information about OAH exposure to organisms within these habitats but provide limited information about the spatial distribution and vertical structure of OAH and are often missing other ecologically relevant parameters such as inorganic nutrients and a second carbonate chemistry parameter^36^. These additional properties are important to develop robust predictive capabilities of OAH and the potential impacts.

Here we report monthly observations of OAH parameters based on measurements of the complete inorganic carbon system coupled with oxygen and dissolved inorganic nutrients along a vertical transect extending from the surf zone to the ~ 40 m isobath in La Jolla, in the Southern California Bight (SCB). The measurements were made between March 2017 to September 2018. These observations fill an important data gap in our perspective of the natural OAH conditions, variability, and distribution in the ecologically important nearshore environment, a need that was recently highlighted in the NOAA ocean acidification research plan for the U.S. West Coast^37^. Specifically, this study addresses three core questions in the SCB nearshore environment: (1) What are the seasonal and spatial ranges of biogeochemical parameters?; (2) What are the pH, Ω_Ar_, and DO minima at different depths?; and (3) What are the frequencies of OAH conditions exceeding geochemical and physiological thresholds at different depths?

## Methods

### Site description

Discrete seawater samples were collected monthly from March 2017 to September 2018 at four stations equally spaced along a transect extending from the nearshore (~ 7 m) to the ~ 40 m isobath located ~ 1.5 km offshore (Supplementary Fig. S1). The transect was located just south of the Ellen Browning Scripps Pier in La Jolla, CA, USA (32.8634° N, 117.2546° W) within the Matlahuayl State Marine Reserve (Supplementary Fig. S1). The benthos underlying the monthly transect is dominated by gradually sloping sand flats with small amounts of macroalgae^38,39^. The transect is also close to the dense *Macrocystis pyrifera* kelp forest that extends up to 8 km southward and 1.5 km offshore^31,40^ (Supplementary Fig. S1). Importantly, the area at large is influenced by the presence of two nearshore submarine canyons, which may funnel deeper water with lower temperature, DO, and pH into the study area^41–43^.

### Sample collection and analysis

**Figure 1:**
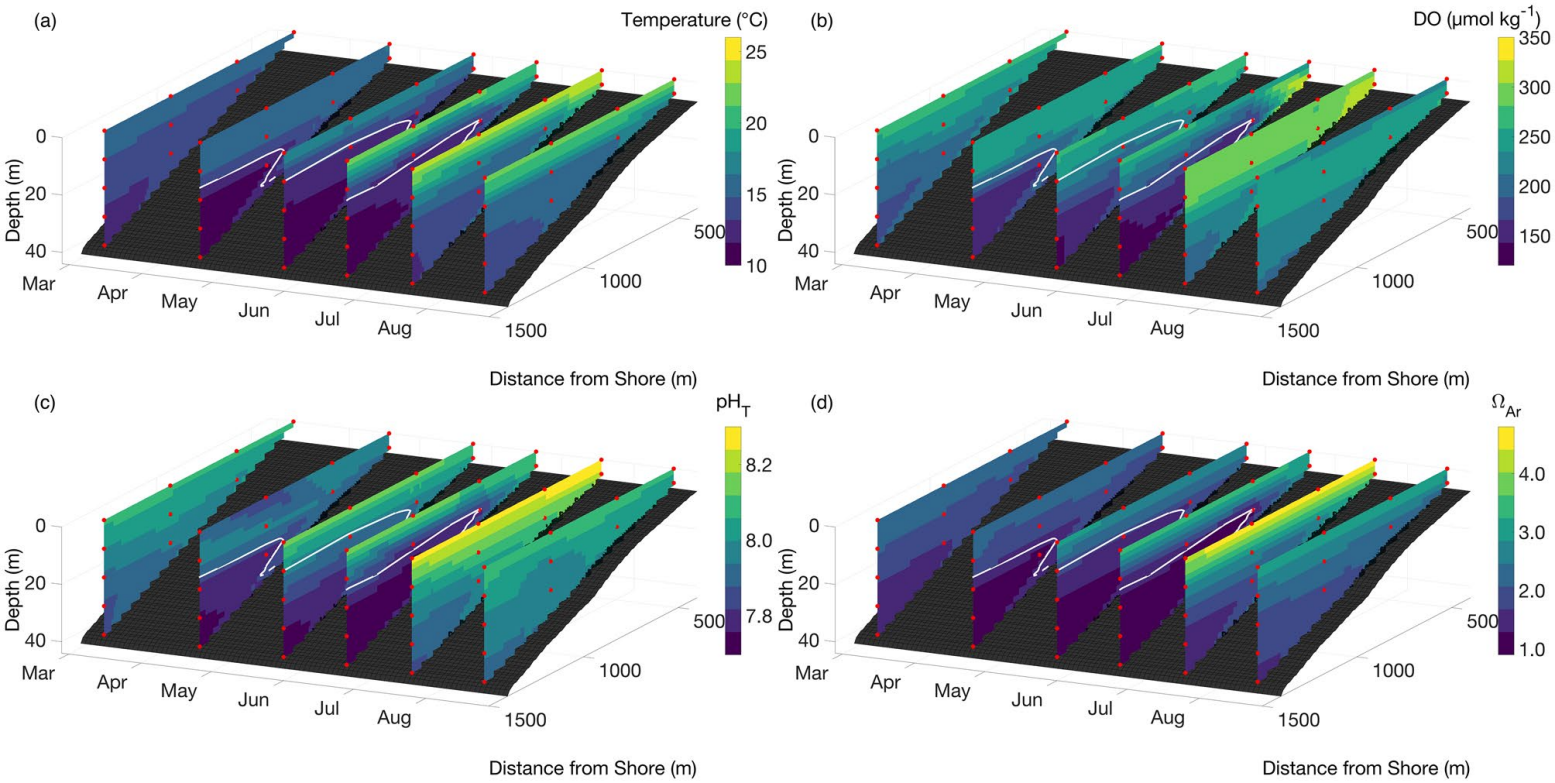
Contours of the spatial and vertical distribution of seawater temperature (**a**), DO (**b**), pH_T_ (**c**), and Ω_Ar_ (**d**) in the nearshore environment in La Jolla, CA, USA between March and August 2017 to highlight changes associated with the upwelling season. Each slice illustrates a single transect with red symbols representing discrete sample depths. The white contour line shows the 25.0 kg m^−3^ σ_θ_ isopycnal.

Seawater samples were collected for quantification of temperature, salinity, DO, DIC, pH_T_ (defined on the total [H^+^] scale), total alkalinity (TA), Ω_Ar_, and dissolved inorganic nutrients (ammonium [NH_4_^+^], nitrate + nitrite [NO_3_^−^ + NO_2_^−^], phosphate [PO_4_^3−^], and silica [SiO_4_]) using a 5 L Niskin bottle. Samples were collected at 10 m depth intervals from the surface to the bottom at each station with the exception of the station most proximal to shore where the bottom sample was collected at 5 m depth (Fig. 1). Samples for seawater carbonate chemistry were collected from the Niskin bottle following standard sampling protocol in 250 mL Corning Pyrex glass bottles and immediately preserved with 100 μL saturated mercuric chloride (HgCl_2_) solution^44^. Inorganic nutrient samples were filtered through 0.45 μm Millipore polycarbonate filters and collected in 30 mL Falcon tubes after three rinses and kept frozen until analysis. Following seawater collection, measurements of temperature (± 0.3 °C), DO (± 2%), and salinity (± 0.1) were taken in the Niskin bottle using a YSI Professional handheld multiparameter instrument (YSI Pro2030). This sampling scheme in theory allows for small seawater heating and uptake of oxygen if DO < 100%, although efforts were made to minimize these effects by maintaining the largest possible volume of water in the Niskin bottle and completing measurements within a few minutes. No differences outside of the precision of the instrument were detected between temperature measurements in the Niskin bottle and direct measurements using a Castaway profiler, but no independent validations exist for the DO measurements. Salinity for the YSI was calibrated to Dickson Certified Reference Material and DO was calibrated in 100% water-saturated air at the start of each transect.

Samples were analyzed for DIC, TA, and pH_T_ in the Scripps Coastal and Open Ocean Biogeochemistry lab. DIC was measured via an automated infrared inorganic carbon analyzer (AIRICA, Marianda) with a LI-COR 7000 as the detector. TA was measured via a potentiometric open-cell acid titration system developed by the laboratory of A. Dickson at Scripps Institution of Oceanography (SIO)^44^. Spectrophotometric pH_T_ was measured with purified metacresol purple dye in a SAMI Autonomous Flow Through pH Instrument (AFT-pH, Sunburst) modified to analyze bottle samples. Accuracy and precision of seawater DIC (0.45 ± 1.57 µmol kg^−1^, n = 69), TA (0.93 ± 1.40 µmol kg^−1^, n = 40), and pH_T_ (± 0.02 for 9 consecutive measurements at 20 °C) were determined with respect to certified reference materials and TRIS buffer solutions provided by the laboratory of A. Dickson.

Dissolved inorganic nutrient samples were analyzed via a Seal Analytical continuous-flow AutoAnalyzer 3 (AA3)^45^ at the Oceanographic Data Facility at SIO using a spectrophotometer to analyze [NO_3_^−^] (± 0.05 µmol kg^−1^), [NO_2_^−^] (± 0.05 µmol kg^−1^), [PO_4_^3−^] (± 0.004 µmol kg^−1^), and [SiO_4_] (± 2 µmol kg^−1^) and a fluorometer to analyze [NH_4_^+^] (± 0.03 µmol kg^−1^) according to standard protocols^46^.

### Data analysis

The MATLAB version of CO2SYS (v2.1)^47^ was used to calculate the seawater aragonite saturation state (Ω_Ar_) using in situ seawater temperature, salinity, TA, and DIC as inputs. K_1_ and K_2_ dissociation constants from Mehrbach et al. (1973) refit by Dickson and Millero (1987), KHSO_4_ dissociation constants from Dickson, and [B]_T_ from Uppstrom (1974) were used for the calculations. Potential density (ρ_θ_) was calculated using measured temperature and salinity using the MATLAB Gibbs SeaWater Oceanographic Toolbox of TEOS-10^48^. σ_θ_ (ρ_θ_ − 1000 kg m^−3^) was used to identify isopycnal surfaces and trace the distribution of water masses through space and time.

To estimate OAH exposure in different habitats, we calculated the frequency distribution of OAH observations for different depths bins (0, 10, 20, 30, and 40 m) based on the data from the first year (i.e., from March, 2017 to February, 2018) at Station 4. The time period was limited to 12 months to avoid uneven weighting by two upwelling seasons. We used Station 4 because there were only small lateral differences between stations and this station represented all depths that were sampled. We also calculated the percentage of DO observations lower than the sublethal hypoxia threshold of ~ 157 µmol kg^−1^ (~ 5 mg L^−1^), a conservative threshold that captures 90% of observed sublethal impacts in temperate benthic marine organisms^49^, and the commonly applied hypoxia threshold of ~ 63 µmol kg^−1^ (~ 2 mg L^−1^)^49,50^. In addition, we calculated the percentage of pH_T_ observations lower than the global mean surface ocean pH_T_ expected by the year 2050 and 2100 (i.e., 7.85 and 7.63) projected by the Coupled Model Intercomparison Project 6 Shared Socioeconomic Pathway-5-8.5 (CMIP6 SSP-5-8.5)^51^, and percentage of Ω_Ar_ observations undersaturated with respect to aragonite (i.e., Ω_Ar_ < 1). Finally, we also compared the observed pH_T_, Ω_Ar_, and DO conditions to experimentally determined sensitivity levels for some organisms found in the SCB that have shown negative responses to these levels^43,52–58^.

## Results

**Figure 2:**
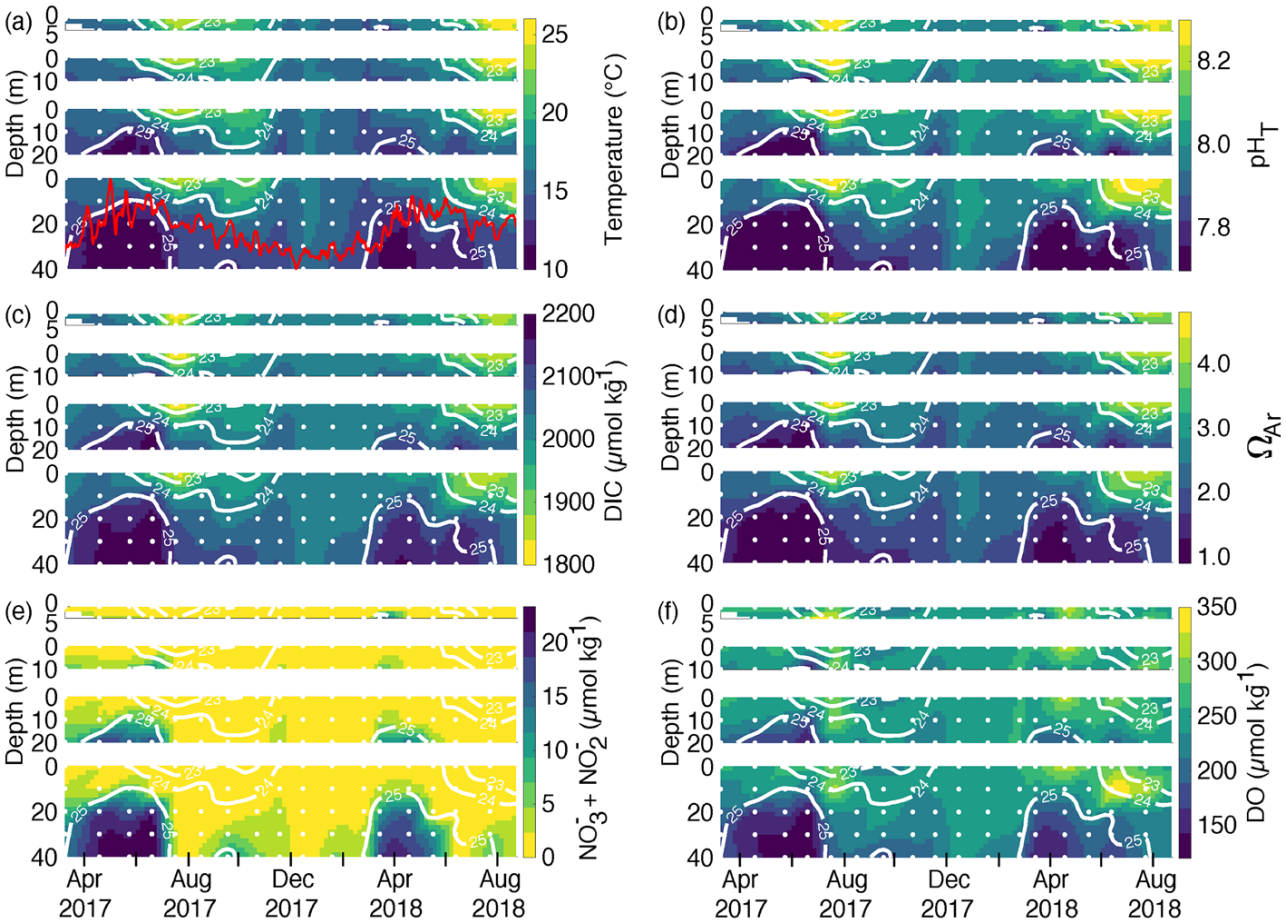
Spatiotemporal contours of seawater temperature (**a**), pH_T_ (**b**), DIC (**c**), Ω_Ar_ (**d**), [NO_3_^−^ + NO_2_^−^] (**e**), and DO (**f**) from March, 2017 to September, 2018. In each panel, the top subplot represents the most nearshore station (Stn 1) while subsequent subplots represent increasing distance from shore (Stn 1–4). White symbols represent discrete seawater samples, the white contour lines represent the 23.0, 24.0, and 25.0 kg m^−3^ σ_θ_ isopycnals, and the red line in panel (**a**) shows the 7-day moving mean of relative upwelling intensity (upwelling intensity/maximum upwelling intensity) based on the Bakun traditional upwelling index at 33° N, 119° W (See Fig. S3 for details).

The nearshore coastal waters exhibited a well-mixed water column with little spatial variability in all measured parameters between December and February. In contrast, during the upwelling season from March to July of both 2017 and 2018, injection of denser seawater with low temperature, pH_T_, Ω_Ar_, DO, and high DIC and dissolved inorganic nutrients (i.e., [SiO_4_], [NO_3_^−^ + NO_2_^−^], and [PO_4_^3−^]) produced strong vertical gradients between the surface and bottom (Figs. 1, 2; Supplementary Fig. S2). The low pH_T_, Ω_Ar_, and DO seawater was roughly constrained by the 25.0 kg m^−3^ isopycnal surface and was observed to intrude to depths as shallow as 10 m and as close as 700 m to the shoreline (Figs. 1, 2). Following the intrusion of the cold, dense, and nutrient enriched seawater, the upper surface layer down to 10 m depth was characterized by high temperature, pH_T_, Ω_Ar_, DO, and low DIC and nutrients between June and August in both 2017 and 2018 (Fig. 2; Supplementary Fig. S2). Salinity and TA displayed slightly elevated values coincident with the upwelling season while [NH_4_^+^] showed no clear seasonal trend throughout the study period (Supplementary Fig. S2). Overall, the general trends in physical and biogeochemical parameters were similar between 2017 and 2018, although the magnitude of both average and extreme values, and timing of deep-seawater intrusion were slightly different (Fig. 2; Supplementary Tables S1, S2; Supplementary Fig. S2). For example, qualitatively lower average values of pH_T_, Ω_Ar_, and DO, were documented between March and July in 2017 compared to 2018 (Supplementary Tables S1, S2).

In comparison to the large vertical gradients in OAH parameters observed during the upwelling season, lateral variability across depth for a given transect during this time was small. For example, the mean (± SD) lateral variability (pH_T_ = 0.03 ± 0.02, Ω_Ar_ = 0.16 ± 0.09, DO = 13 ± 11 µmol kg^−1^) was much smaller compared to the mean (± SD) vertical gradients (pH_T_ = 0.25 ± 0.13, Ω_Ar_ = 1.56 ± 0.81, DO = 90 ± 55 µmol kg^−1^) by a factor of 7 to 10 for pH_T_, Ω_Ar_ and oxygen.

**Figure 3:**
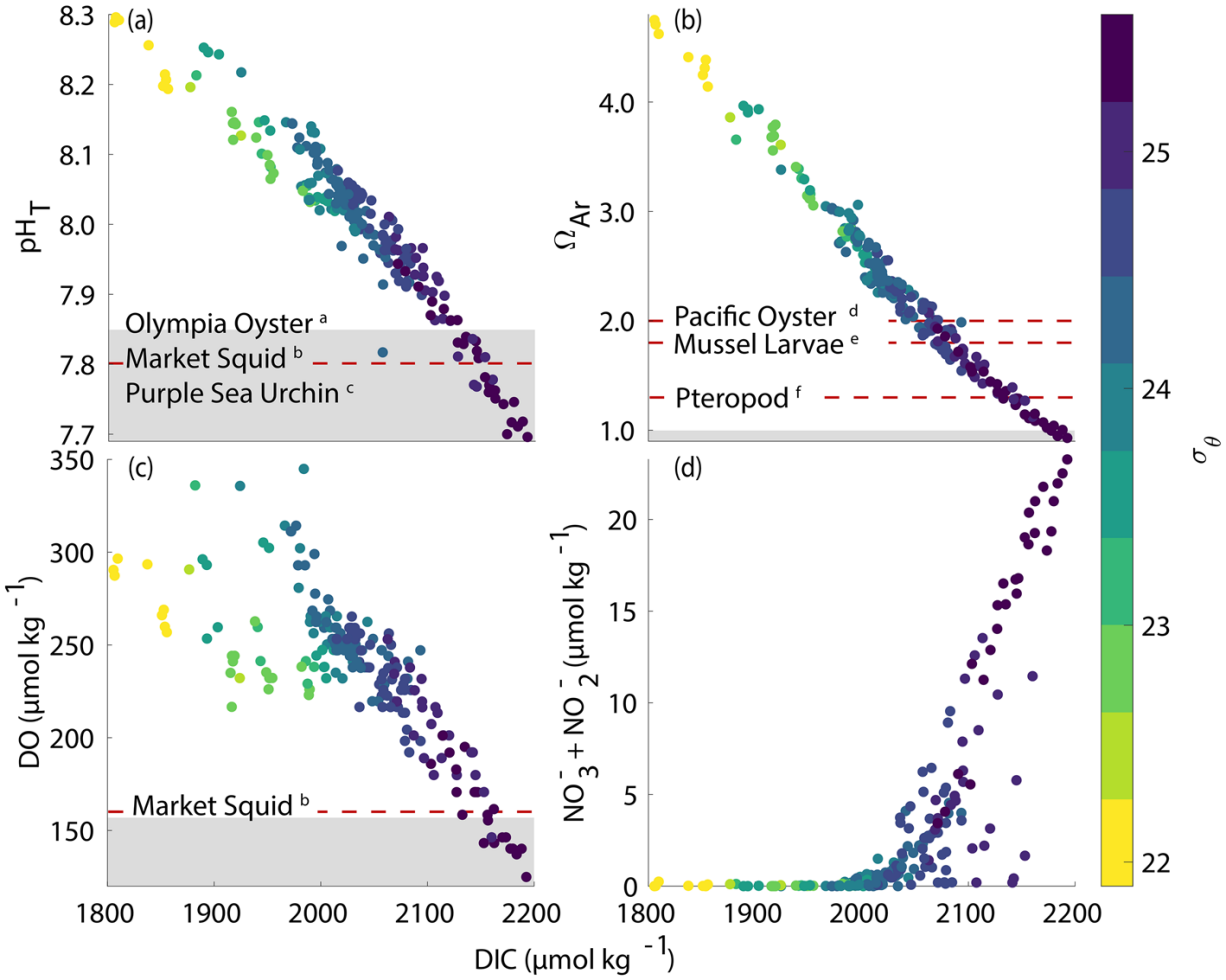
Property-property plots of pH_T_, Ω_Ar_, DO, and [NO_3_^−^ + NO_2_^−^] as a function of DIC colored by seawater σ_θ_ from all discrete seawater samples. Grey regions in a, b, and c signify mid-century mean oceanic pH_T_ = 7.85 for SSP-5-8.5 (**a**), Ω_Ar_ < 1 (**b**), and sublethal hypoxic threshold, DO < 157 µmol kg^−1^ (**c**). Red-dashed lines indicate experimentally observed sensitivity levels for the *a*: Olympia oyster^52^, *b*: market squid^43^, *c*: purple sea urchin^53–55^, *d*: Pacific oyster^58^, *e*: California mussel larvae^57^, and *f*: pteropods^56^, i.e., at these levels, some form of negative effect on the organisms fitness was documented.

The lowest values of pH_T_ (7.69), Ω_Ar_ (0.93), and DO (125 µmol kg^−1^) occurred during the upwelling season at the deepest depth (40 m). However, pH_T_, Ω_Ar_, and DO values as low as 7.77, 1.12, and 146 µmol kg^−1^, respectively, were observed at depths as shallow as 10 m (Fig. 2). The minimum values of these parameters at depth coincided with the highest density (σ_0_ = 25.6 kg m^−3^), lowest temperature (10.5 °C), highest DIC (2193 µmol kg^−1^), and highest inorganic nutrient concentrations ([SiO_4_] = 23.9 µmol kg^−1^, [NO_3_^−^ + NO_2_^−^] = 23.9 µmol kg^−1^, and [PO_4_^3−^] = 1.9 µmol kg^−1^) (Fig. 2; Supplementary Fig. S2) and strong correlations indicative of upwelled deep water were observed for the key biogeochemical parameters. In seawater characterized by σ_0_ > 25.0 kg m^−3^, pH_T_, Ω_Ar_, and DO showed strong, inverse, linear correlations with DIC whereas [NO_3_^−^ + NO_2_^−^] showed a strong positive correlation with DIC (Fig. 3). Although correlations among these parameters were also observed in less dense water, the spread of data typically increased and the slope of the relationship between each parameter and DIC also changed.

**Figure 4:**
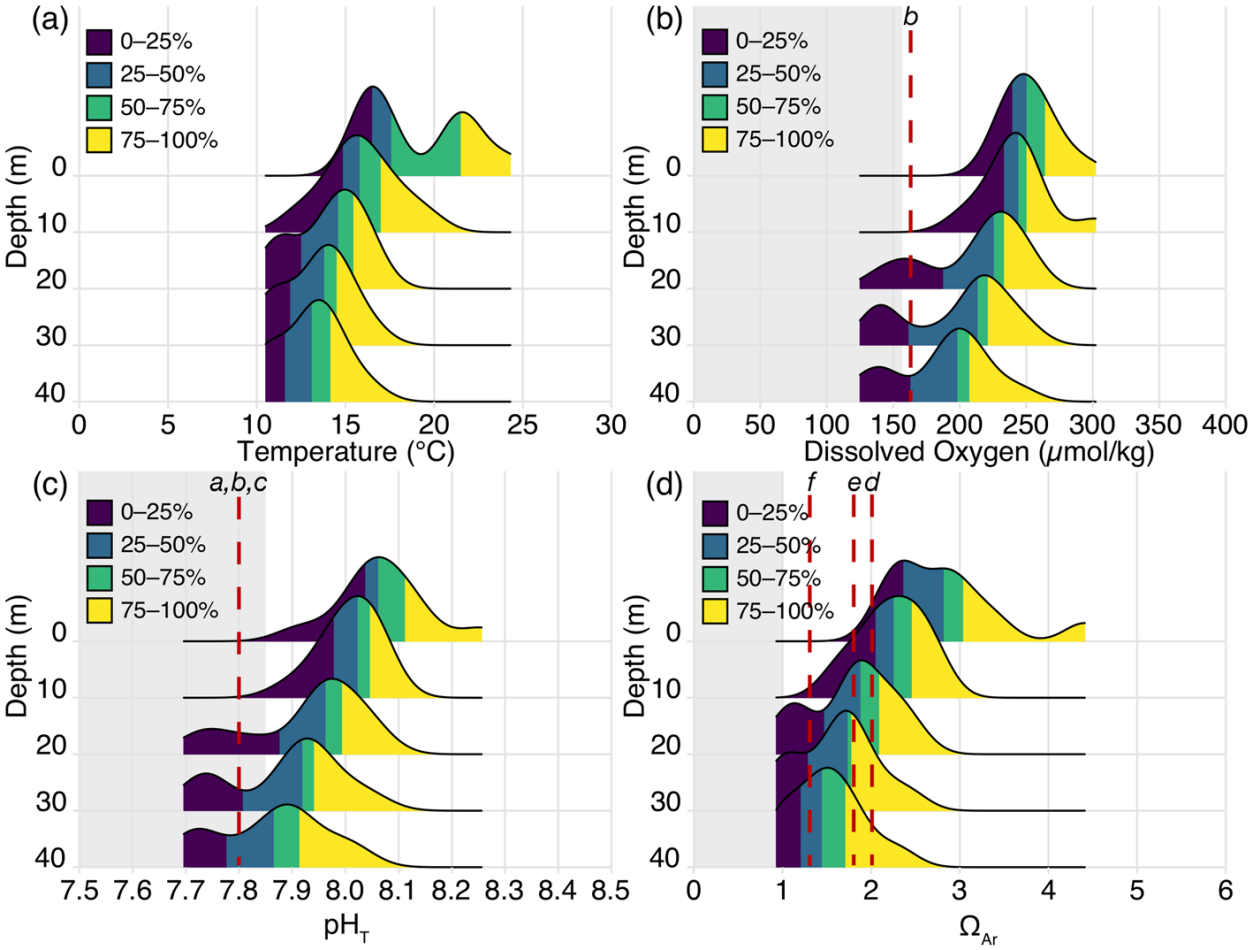
Density ridgeline plots showing frequency distribution of observations of seawater temperature (**a**), DO (**b**), pH_T_ (**c**), and Ω_Ar_ (**d**) at the 0, 10, 20, 30, and 40 m depth bins at Station 4 over the first year of the study. Percentages of observations are displayed in quartiles. Grey regions on panels **b**, **c**, and **d** signify sublethal hypoxic threshold (DO < 157 µmol kg^−1^), mid-century mean oceanic pH_T_ = 7.85 for SSP-5-8.5, and Ω_Ar_ < 1, respectively. Red-dashed lines indicate experimentally observed sensitivity levels for regional fauna as described in Fig. 3 (*a*: Olympia oyster^52^, *b*: market squid^43^, *c*: purple sea urchin^53–55^, *d*: Pacific oyster^58^, *e*: California mussel larvae^57^, and *f*: pteropods^56^).

The frequency distributions of temperature, pH_T_, Ω_Ar_, and DO observations from Station 4 at different depths expressed as quartiles (i.e., 0–25%, 25–50%, 50–75%, 75–100%), shifted towards lower values at deeper depths (Fig. 4). Seawater temperature and Ω_Ar_ were slightly bimodal and skewed towards higher values at the surface and 10 m depth but became more uniformly distributed below this depth (Fig. 4). Seawater Ω_Ar_ at the surface and at 10 m depth was never undersaturated while 8% of the observations below 20 m depth were undersaturated (Ω_Ar_ < 1) (Fig. 4). pH_T_ and DO showed different distributions compared to seawater temperature and Ω_Ar_ with a more uniform distribution at the surface becoming bimodal and increasingly skewed towards lower values at depth (Fig. 4). 28% of pH_T_ observations at 20–40 m depths were lower than 7.85, the CMIP6 SSP-5-8.5 surface ocean projections anticipated by 2050^51^. No pH_T_ observations were less than 7.63, the mean surface ocean pH_T_ anticipated by the year 2100 under SSP-5-8.5. 19% of DO observations were lower than the sublethal threshold of 157 µmol kg^−1^^49^, but there were no DO observations below 63 µmol kg^−1^ (Fig. 4). Based on experimentally derived sensitivities to pH and/or DO for organisms present in the SCB (i.e., Olympia oyster^52^, market squid^43^, purple sea urchin^53–55^), their sensitivity levels were crossed in up to 25% of the observations below 20 m depth (Fig. 4). Similar results were observed for Ω_Ar_ sensitivity derived for pteropods^56^ (Ω_Ar_ = 1.3) while Ω_Ar_ sensitivity for the mussel larvae^57^ (Ω_Ar_ = 1.8) and Pacific oyster^58^ (Ω_Ar_ = 2.0) were crossed 50–75+% at depth below 20 m and up to 25% at 10 m depth.

## Discussion

### Seasonal variability

The largest variability and most intense values of OAH parameters in this nearshore SCB ecosystem were clearly associated with seasonal wind-driven upwelling, which generally begins in March and reaches maximum intensity between May and July^23–25^ (Fig. 2; Supplementary Figs. S3, S4, S5). The occurrence of cold, dense, nutrient-rich, low pH_T_, Ω_Ar_, and DO seawater observed in the nearshore environment during this period clearly reflected a deeper origin, but the exact trajectory and how long the transit from deep to shallow took is unknown. Seawater with a distinct upwelling signature was typically associated with a σ_θ_ equal to or greater than 25.0 kg m^−3^. Time-series observations from the nearby Del Mar Mooring, located at the shelf-break (Supplementary Fig. S1), corroborate these observations and show that the seasonal shoaling of isopycnals at this location roughly tracks the upwelling intensity (Supplementary Fig. S4). Seaward of the shelf-break, the 25.0 kg m^−3^ isopycnal is typically found at depths of approximately 50–80 m outside of the upwelling season^9,42,59^. Comparison with data from years prior to and following this study (2015–2021) provide no obvious indications that 2017 and 2018 were radically different from these other years (Supplementary Fig. S4).

Concurrent with the peak of the upwelling season, observations of high pH_T_, Ω_Ar_, and DO in the surface seawater between June and August resulted from a phytoplankton bloom presumably fueled by the intrusion of nutrients that coincided with sea surface warming and high light availability. Similar to the upwelling dynamics, this bloom is an annual occurrence that is evident in historical chlorophyll records from the SIO Pier^60^ and from region-wide ocean color data from the SCB (Supplementary Fig. S6). Notably, the phytoplankton bloom exacerbated vertical gradients in pH_T_ and oxygen due to photosynthesis in the surface layer.

Although the general trends and timing in OAH parameters were similar between the upwelling seasons of 2017 and 2018, lower OAH values were observed in 2017 (Supplementary Tables S1, S2). This is likely associated with differences in the timing, intensity, and source of upwelled seawater between the 2 years^27,61,62^ (Supplementary Fig. S3), but could also be a misleading representation due to the monthly sampling scheme that overlooks variability at higher frequencies. Previous studies within the study area have demonstrated that high-frequency intensification and relaxation of upwelling occurs throughout the upwelling season with short-term exposure to extreme OAH conditions of < 1 week^31,34,42,43^. Future studies would benefit from a combination of high-frequency autonomous measurements at select locations and the sampling scheme employed here to more fully elucidate the nearshore biogeochemical spatiotemporal variability in relation to variability in upwelling intensity and the meteorological forcings.

Irrespective of the temporal limitations of the monthly data record, the most intense signal and lowest values of pH, Ω_Ar_, and DO were observed between May and June of both years and coincided with the peak upwelling intensity (Fig. 2; Supplementary Fig. S3). Furthermore, the most intense pH, Ω_Ar_, and DO values were always observed at the deepest depth and in the seawater with the highest density (Figs. 1, 2, 4), indicating a clear link between the OAH intensity and the source water. Because of the seasonal occurrence of upwelling combined with different OAH properties between upwelled water and the surface mixed layer, OAH parameters tended to follow a skewed bimodal frequency distribution as a function of depth on an annual basis (Fig. 4). That is, in the absence of upwelling, each parameter mostly followed a normal distribution with decreasing mean values with increasing depth. Because of the seasonal upwelling and injection of seawater with lower pH_T_, Ω_Ar_, and DO, this caused a second peak in the frequency distribution towards lower values for each parameter giving rise to a bimodal left skewed distribution on an annual basis. The fact that observations at depths greater than 20 m showed consistent occurrences of seawater pH_T_ lower than levels anticipated in the surface ocean by 2050 (under SSP-5-8.5)^51^, undersaturation with respect to aragonite, and DO levels lower than the sublethal hypoxia threshold demonstrate that marine organisms and ecosystems below this depth may already experience OAH stress^57,52–58^. This may also be inferred from comparison with experimental OAH levels shown to negatively affect local organisms^57,43,52–58^, which were consistently crossed at depths greater than 20 m (Figs. 3, 4). However, because of an absence of historical observations and large amount of natural variability in the nearshore SCB, we cannot rigorously determine to what extent the current conditions are outside of the pre-industrial envelope of variability. A previous study in the SCB estimated based on in situ measurements and model calculations that the annual mean seawater pH in the surface to 30 m depth has declined by 0.13–0.14 units while Ω_Ar_ has declined by 0.64–0.71 units since preindustrial time^34^. Nonetheless, because of numerous assumptions and differential signatures of anthropogenic carbon between the surface mixed layer and deeper water masses, some caution is advised in accepting this level of anthropogenic modification in the most intense pH and Ω_Ar_ values documented here during the upwelling season. Regardless of these previous findings, in terms of organismal impacts, it is clear that the seasonal and interannual variability in seawater pH and Ω_Ar_ due to upwelling is currently more critical to consider than the long-term secular change in these parameters owing to anthropogenic carbon uptake. The secular anthropogenic signal will become increasingly important on timescales of decades and longer, but it is important to view this in the context of the seasonal and interannual variability, and not only by the annual mean surface ocean pH and Ω_Ar_ as highlighted by the results in this study.

### Potential impacts on marine organisms

Numerous studies have categorized the organisms found in the nearshore environment of the SCB, which include many economically and ecologically important taxa such as spiny lobsters, sea urchins, squids, kelp, and seagrass (e.g.,^43,52–55,63–66^). Many of these organisms occur at depths exceeding 20 m^65^, and are thus exposed to the OAH conditions documented here, but it is unknown whether these conditions have any direct negative impacts on these organisms in their natural environment at this time. Based on experimental assessments of the sensitivity to OAH for some of the organisms common in this region, the observed conditions could be detrimental to some aspect of their fitness. For example, experiments with Olympia oyster and California purple sea urchin larvae showed decreased growth rates at pH_T_ ≤ 7.80^52–55^, spiny lobsters from La Jolla showed decreased ability to detect chemical cues at pH_T_ = 7.67^66^, and the market squid might experience temporal habitat compression due to shoaled seawater with pH_T_ < 7.80 and DO < 160 µmol kg^−1^^43^. pH_T_ and DO levels less than these values were documented for extended periods during the upwelling season at depths greater than 20 m. Similarly, seawater aragonite saturation state lower than experimentally documented levels of sensitivity for Pacific oysters (Ω_Ar_ = 2.0)^58^, California mussel larvae (Ω_Ar_ = 1.8)^57^, and pteropods (Ω_Ar_ = 1.3)^56^ were observed at depths as shallow as 10 m (Fig. 4). However, it is important to note that the observed OAH conditions are accompanied by nutrients that fuel the high productivity in this region and could potentially compensate for any negative effects arising from OAH as it has been well established that many organisms are less vulnerable to environmental stress given plentiful access to nutrition and energy^67,68^. Furthermore, many marine organisms in La Jolla are associated with the existing kelp forests, which are found in the depth range of 10–30 m (Supplementary Fig. S1). Kelp forests have been hypothesized as potential refugia against ocean acidification^31,69,70^ due to their ability to locally elevate seawater pH_T_ and their own resilience to elevated pCO_2_ and low pH_T_^71–73^. Additionally, kelps are predicted to benefit from hypoxia due to decreased predation by grazers^73^, and the abundance of kelp is strongly dependent on a sufficient supply of nitrate associated with deeper water^40^. It is evident that additional research is required to elucidate the potential impacts on organisms in the context of the chemical conditions documented in this study. This includes a better understanding of the combined influence of low pH and high nutrient conditions on organisms as well as the influence from kelp.

### Future implications

Future projections suggest that OAH conditions in the California Current Ecosystem are likely to be exacerbated owing to a combination of intensifying upwelling^26,74^, deeper source water contribution^61,62^, longer duration of upwelling^74^, and a gradually increasing anthropogenic signal^34^, but there are large uncertainties associated with exactly what these scenarios will look like. This is partly due to our limited understanding of present-day conditions and variability, the underlying drivers, and exactly how the governing processes will change under a changing climate. For example, will the OAH conditions characterized in this study remain at depth > 20 m or will they gradually reach shallower environments and persist for longer durations? Furthermore, given that the upwelled pH and Ω_Ar_ levels are a complex function of the upwelling depth, source water, surface productivity and subsequent remineralization, elemental stoichiometry, and ocean currents^75–77^, to what extent will these properties change?

Numerical models may offer the best approach to address these questions, but most existing models are currently too coarse to resolve the spatiotemporal biogeochemical variability documented in this study. Existing models typically range in horizontal resolution from 1 to 4 km or larger (e.g.,^22,76,78–82^) and therefore fail to reveal the full extent of ecologically relevant OAH dynamics in the proximal nearshore environment due to spatial and temporal averaging (e.g.,^22^). However, there is an increasing recognition that higher model resolution is required to accurately reproduce and forecast biogeochemical and biological impacts of climate change in this region as important differences have emerged in down-scaled model simulations^83^. A recent modeling effort of the SCB has a horizontal resolution of 0.3 km^84^, which offers potential for comparison with the observational data of the current study. Currently, seawater carbonate chemistry parameters of this model are validated by CalCOFI data collected 3–10 km from shore, which do not capture the shallow nearshore dynamics demonstrated here, but the potential for synergy between such a model and nearshore high resolution observations is evident.

In summary, this study shows the progression and evolution of OAH parameters over two upwelling seasons along a vertical transect in the nearshore environment of SCB. The observed magnitude and annual frequency of extreme OAH conditions in the depth range 20–40 m raise concerns about the potential effects on organisms and how future intensified upwelling conditions may exacerbate these conditions. At the same time, organisms’ windows of tolerance, acclimatization, and adaptation potential are currently weakly constrained in relation to the documented OAH and nutrient dynamics associated with upwelling, and most models are currently too coarse to precisely model the fine scale dynamics of the shallow nearshore environment. Interdisciplinary efforts combining chemical and biological observations, experiments, and numerical model simulations would be advantageous for promoting rapid scientific advancement on this topic.

## Supplementary Information

Supplementary Information.

## Data Availability

Data analyzed for this study are available at BCO-DMO https://www.bco-dmo.org/dataset/839175.
