# Supplementary material for: Seasonal nearshore ocean acidification and deoxygenation in the Southern California Bight
Source: Sci Rep. 2022 Oct 26;12:17969. doi: 10.1038/s41598-022-21831-y (PMC9606271; doi:10.1038/s41598-022-21831-y)
Supplement: Supplementary file 1 — Supplementary Information. [file 41598_2022_21831_MOESM1_ESM.docx]

Seasonal near-shore ocean acidification and deoxygenation in the Southern California Bight

Samuel A. H. Kekuewa,^1^ Travis A. Courtney,^2^ Tyler Cyronak,^3^ Andreas J. Andersson^1^

^1^ Scripps Institution of Oceanography, University of California San Diego, La Jolla, California, USA,

^2^ Department of Marine Sciences, University of Puerto Rico Mayagüez, Mayagüez, Puerto Rico,

^3^ Department of Marine and Environmental Sciences, Halmos College of Natural Sciences and Oceanography, Nova Southeastern University, Dania Beach, Florida

Corresponding authors: Samuel Kekuewa (kekuewa.sam@gmail.com) and Andreas Andersson ([aandersson@ucsd.edu](mailto:aandersson@ucsd.edu))

Supplemental Information


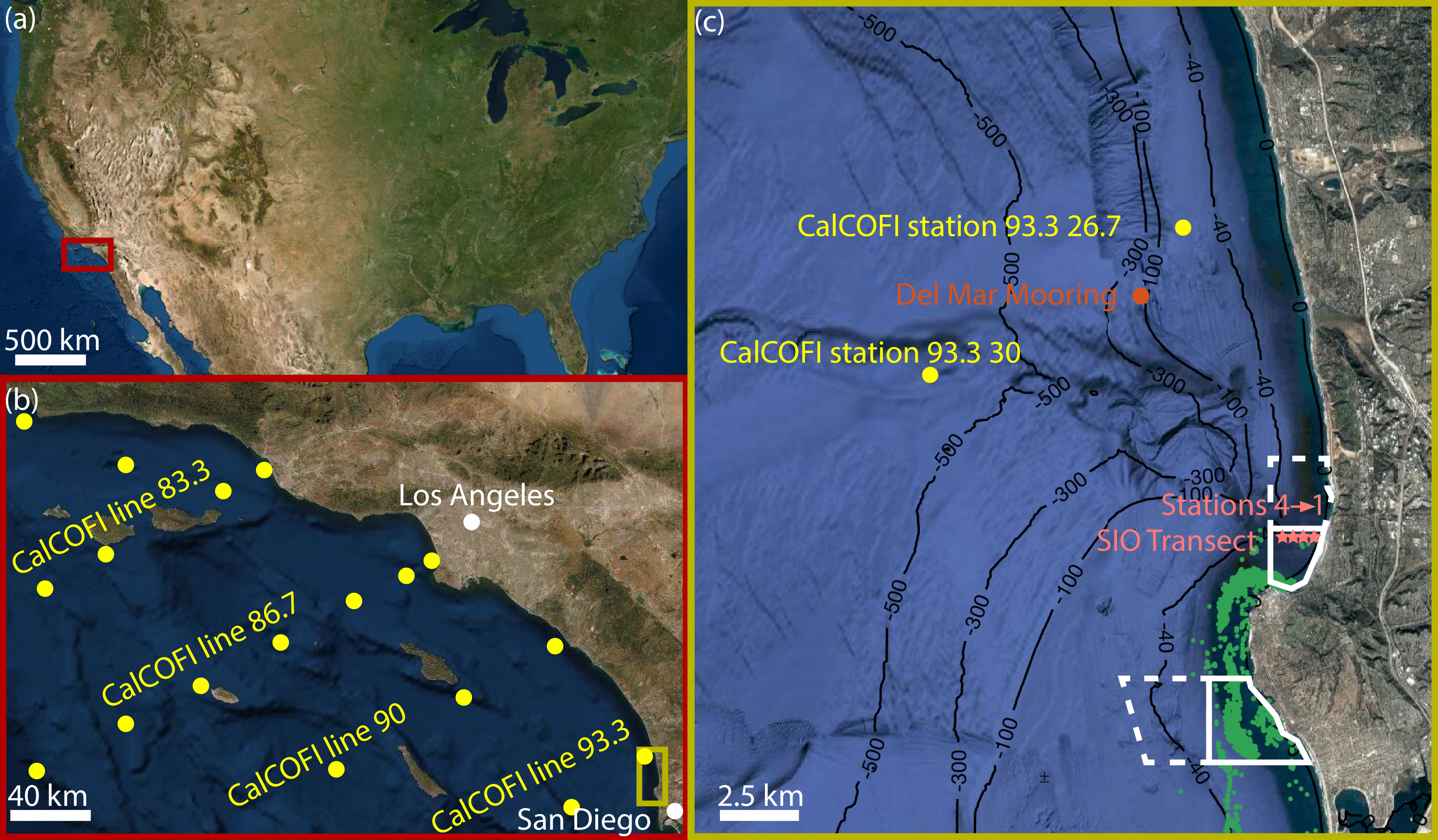


**Figure S1.** Maps of the (**a**) United States, (**b**) Southern California Bight, and (**c**) study location in La Jolla, California. The yellow dots show locations of CalCOFI sampling stations, the orange dot shows the Del Mar Mooring, and the light-red stars show sampling stations 4 to 1 for this study. Overlaid on top of the map in (**c**), the black contours represent the 0, 40, 100, 300, and 500 m depth contours. The green dots illustrate locations of persistent kelp canopy and the white boxes highlight State Marine Reserves (solid-line) and State Marine Conservation Areas (dashed-line) (Data credit: California Department of Fish and Wildlife). Maps were generated in MATLAB using plot_google_map (Zohar Bar-Yehuda (2022). zoharby/plot_google_map (https://github.com/zoharby/plot_google_map), GitHub).


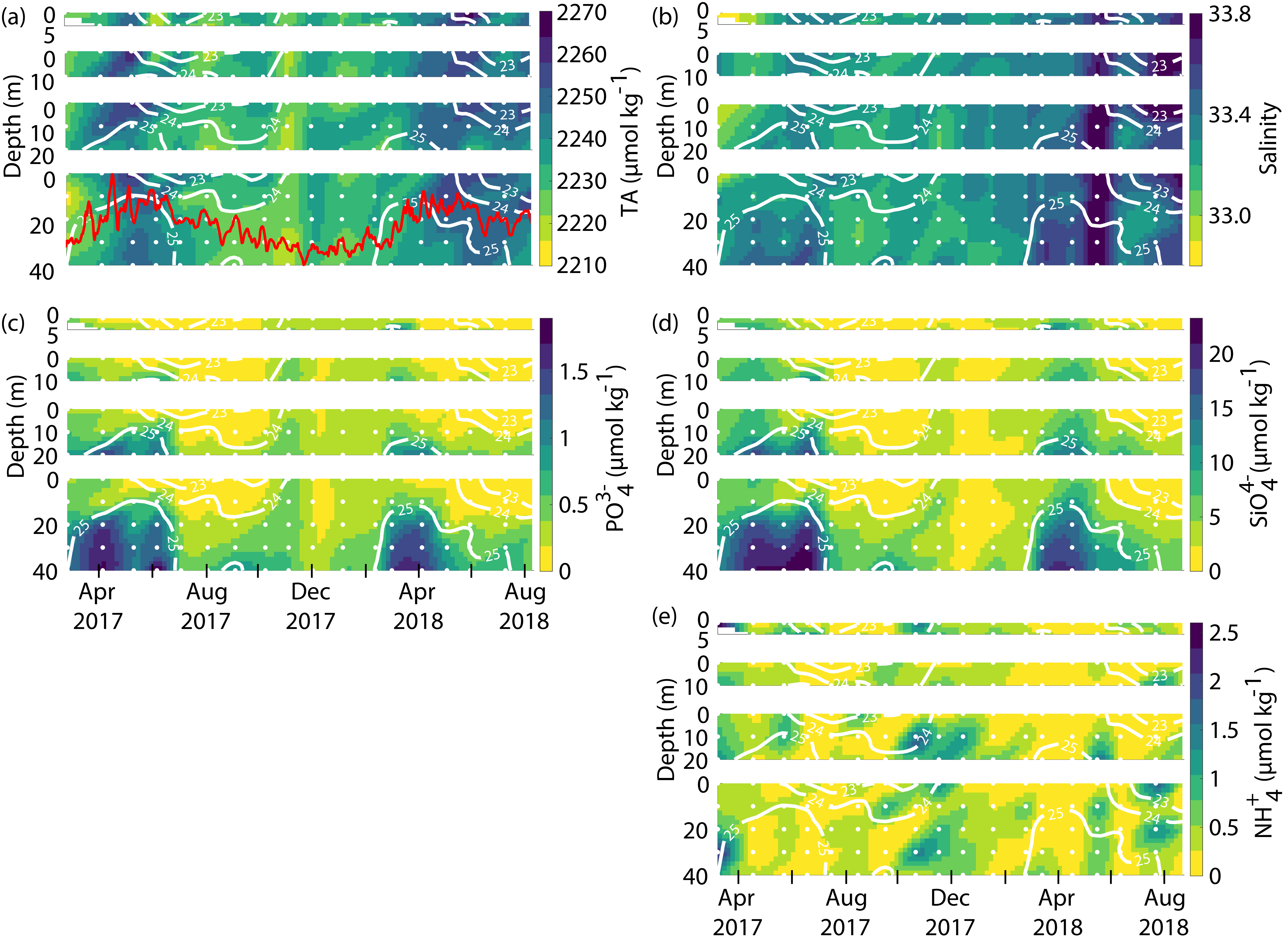


**Figure S2.** Spatiotemporal contour plots of seawater (**a**) total alkalinity (TA), (**b**) salinity, (**c**) [PO_4_^3-^], (**d**) [SiO_4_^4-^], and (**e**) [NH_4_^+^] from March, 2017 to September, 2018 in La Jolla. In each panel, the top subplot represents the most near-shore station (Stn 1) while subsequent subplots represent increasing distance from shore (Stn 1-4). The white symbols represent discrete seawater samples and the white contour lines represent the 23.0, 24.0, and 25.0 σ_θ_ isopycnals, and the red line in panel (**a**) shows the 7-day moving mean of relative upwelling intensity (upwelling intensity/maximum observed upwelling intensity) based on the Bakun traditional upwelling index at 33° N, 119° W (See Fig. S3 for details).


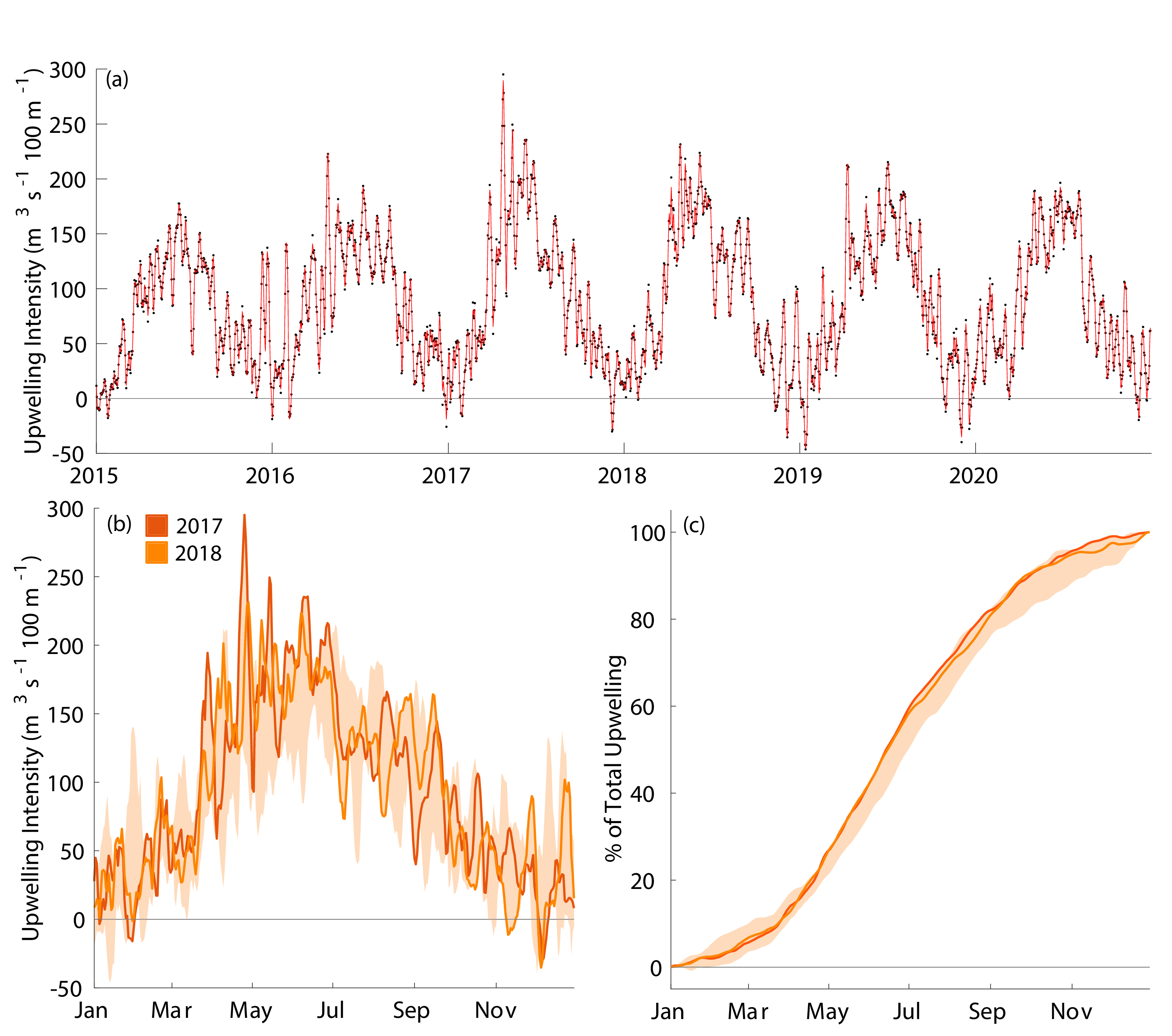


**Figure S3.** (**a**) Black dots represent 7-day moving mean of upwelling intensity (m^3^ s^-1^ 100m^-1^) based on the Bakun traditional upwelling index at 33° N, 119° W from 2015 to 2021 fit to a smoothing spline (red line). The Bakun upwelling index is calculated and provided by the NOAA Environmental Research Division (https://oceanview.pfeg.noaa.gov/products/upwelling/bakun). The index estimates surface transport from pressure fields using the geostrophic wind approximation (for details see link above and references therein). (**b**) Range of upwelling intensity of 7-day moving mean from January to December for the years 2015 to 2021 (shaded area), the dark orange line represents upwelling intensity in 2017 while the lighter orange represents upwelling intensity in 2018. (**c**) Percent of total upwelling experienced by each month based on the same color scheme as in (**b**).


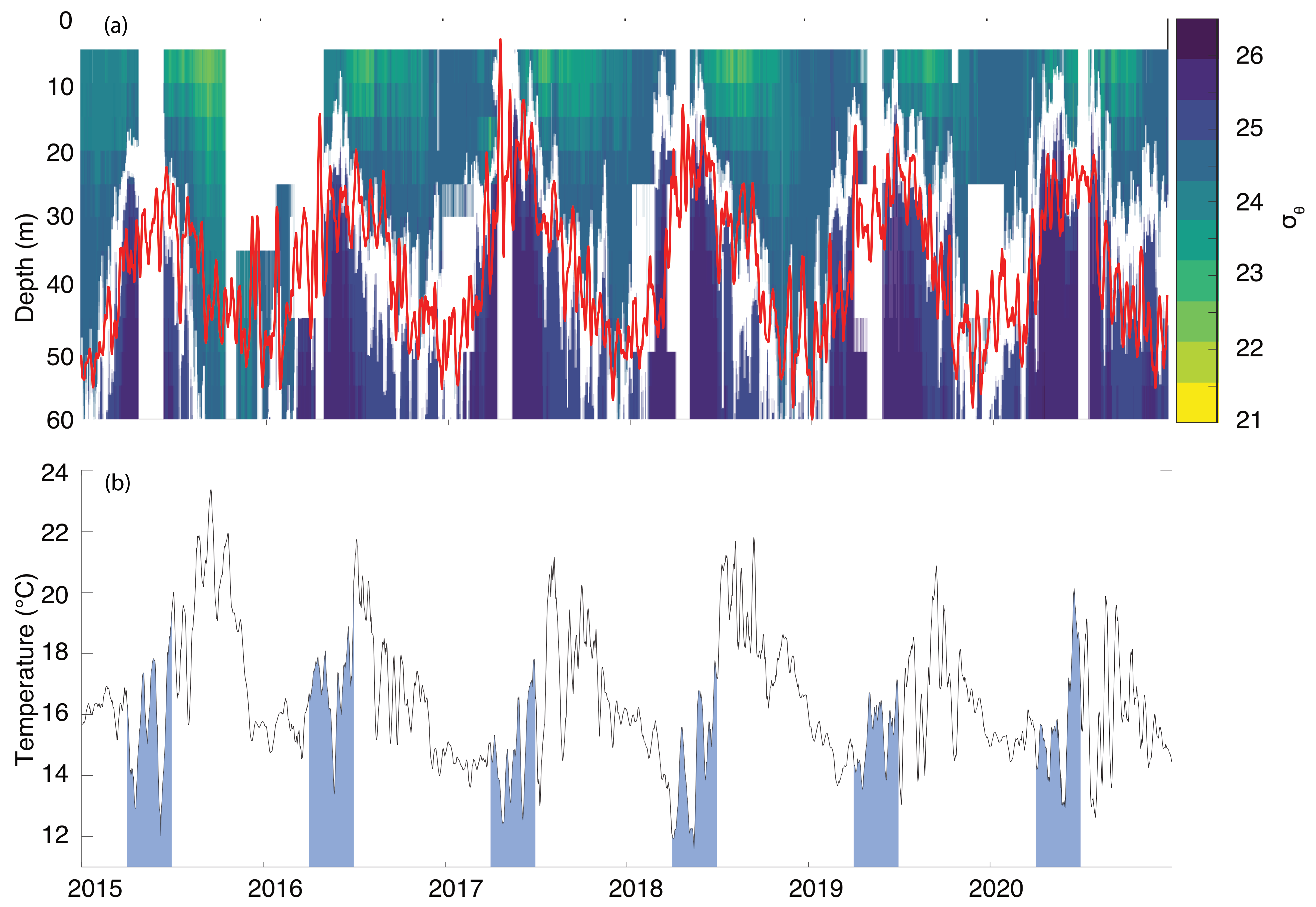


**Figure S4.** (**a**) Upwelling intensity (red line) overlaid spatiotemporal contours of σ_θ_ (kg m^-3^) from January, 2015 to January, 2021 based on measurements from the Del Mar Mooring (Data credit: SIO Ocean Time-Series Group, Prof. Uwe Send). The white line shows the 25.0 σ_θ_ isopycnal. The upwelling intensity is shown as the 7-day moving mean (m^3^ s^-1^ 100m^-1^) at 33° N, 119° W as described in Fig. S3. (**b**) Minimum seawater temperature based on daily surface water measurements from the Ellen Browning Scripps Memorial Pier from January, 2015 to January, 2021 as part of the Southern California Coastal Ocean Observing System shown as a 7-day moving mean (https://data.caloos.org/#metadata/110895/station/data). Blue-shaded areas highlight April 1 – July 1 (i.e., upwelling season) of each year.


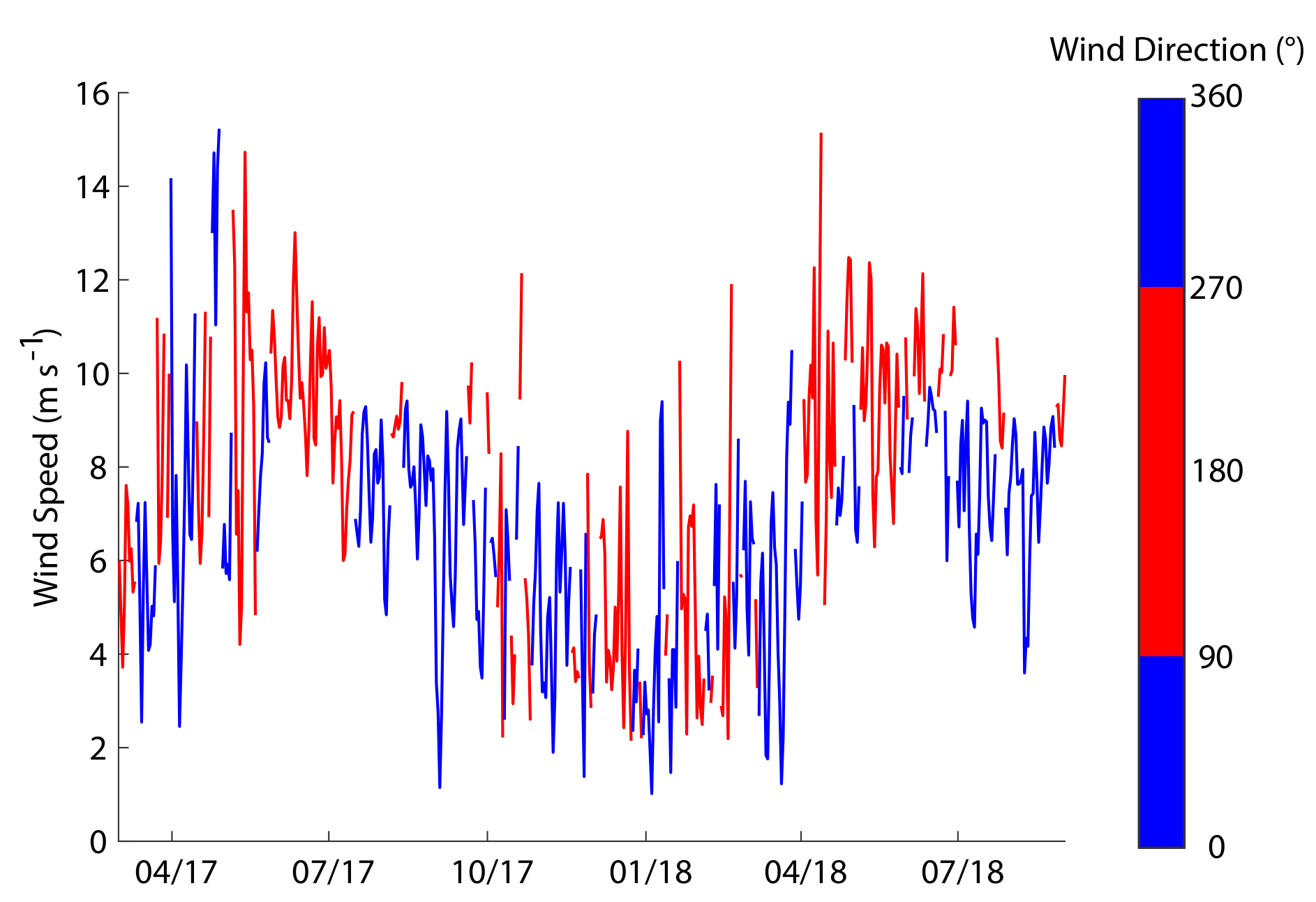


**Figure S5**. 7-day moving mean of wind speed at 33° N, 119° W from March 1, 2017 to October 1, 2018. Wind speed data is colored by wind direction wherein the red from 90° to 270° indicates the Eastward-Southward-Westward directions and the blue from 270° to 90° indicates the Westward-Northward-Eastward directions. Data was based off of six-hourly derived wind data provided by the NOAA Environmental Research Division (https://oceanview.pfeg.noaa.gov/products/upwelling/dnld).


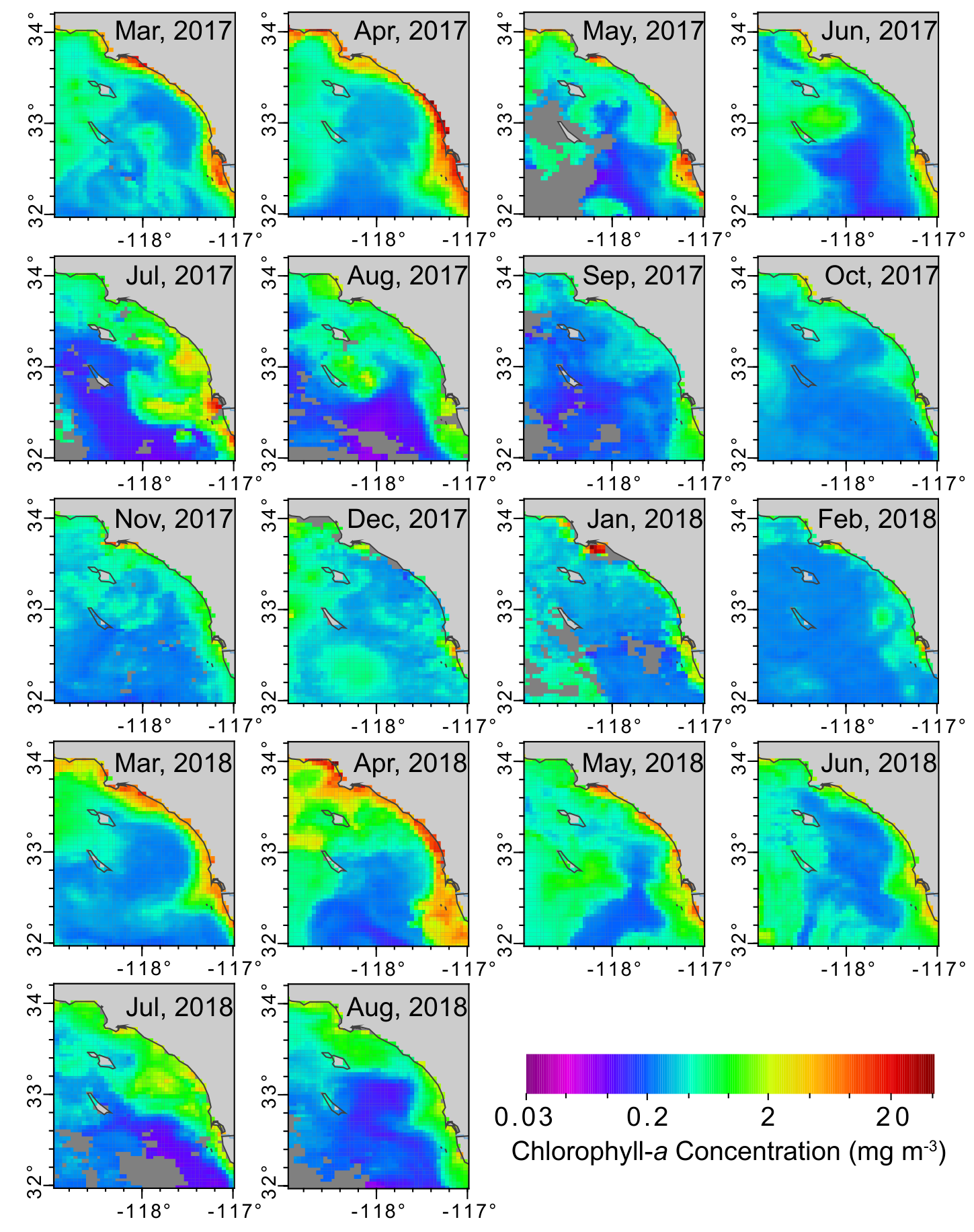


**Figure S6**. Regional 8-day composites of chlorophyll-*a* data from MODIS-Aqua for each month from March, 2017 to August, 2018.

| Table S1. Average ± 1 standard deviation seawater temperature, dissolved inorganic carbon (DIC), pH_T_, aragonite saturation state (Ω_Ar_), dissolved oxygen (DO), and [NO_3_^-^+NO_2_^-^] concentrations from March-July of 2017 and 2018 for each sampling depth at Station 4. | | | | | | |
| --- | --- | --- | --- | --- | --- | --- |
| March-July, 2017 | | | | | | |
| Depth (m) | **Temp**  **(°C)** | **DIC**  **(μmol kg^-1^)** | **pH_T_** | **Ω_Ar_** | **DO**  **(μmol kg^-1^)** | **[NO_3_^-^+NO_2_^-^]**  **(μmol kg^-1^)** |
| 0 | 19.1±3.6 | 1965±83 | 8.10±0.12 | 3.05±0.91 | 267±17 | 0.6±01.0 |
| 10 | 14.6±1.9 | 2058±53 | 7.98±0.06 | 2.04±0.43 | 239±39 | 3.3±2.9 |
| 20 | 12.2±1.4 | 2127±46 | 7.84±0.10 | 1.36±0.35 | 180±36 | 12.8±8.2 |
| 30 | 11.7±1.2 | 2147±39 | 7.79±0.08 | 1.22±0.27 | 162±31 | 15.7±8.5 |
| 40 | 11.5±1.3 | 2157±38 | 7.78±0.08 | 1.17±0.24 | 160±33 | 16.9±8.6 |
| March-July, 2018 | | | | | | |
| Depth (m) | **Temp**  **(°C)** | **DIC**  **(μmol kg^-1^)** | **pH_T_** | **Ω_Ar_** | **DO**  **(μmol kg^-1^)** | **[NO_3_^-^+NO_2_^-^]**  **(μmol kg^-1^)** |
| 0 | 19.5±3.8 | 1944±69 | 8.15±0.08 | 3.32±0.78 | 286±19 | 0.0±0.0 |
| 10 | 16.6±2.7 | 2001±65 | 8.09±0.10 | 2.67±0.66 | 275±44 | 1.7±2.2 |
| 20 | 13.6±1.5 | 2095±29 | 7.93±0.06 | 1.74±0.31 | 213±21 | 5.9±7.0 |
| 30 | 12.5±0.9 | 2125±24 | 7.86±0.06 | 1.47±0.23 | 190±26 | 9.9±7.3 |
| 40 | 12.1±0.9 | 2137±28 | 7.83±0.07 | 1.35±0.25 | 177±28 | 11.7±7.9 |

| Table S2. Observed minimum and maximum (min-max) of seawater temperature, dissolved inorganic carbon (DIC), pH, aragonite saturation state (Ω_Ar_), dissolved oxygen (DO), and [NO_3_^-^+NO_2_^-^] concentrations from March-July of 2017 and 2018 for each sampling depth at Station 4. | | | | | | |
| --- | --- | --- | --- | --- | --- | --- |
| March-July, 2017 | | | | | | |
| Depth (m) | **Temp**  **(°C)** | **DIC**  **(μmol kg^-1^)** | **pH_T_** | **Ω_Ar_** | **DO**  **(μmol kg^-1^)** | **[NO_3_^-^+NO_2_^-^]**  **(μmol kg^-1^)** |
| 0 | 16.1-24.3 | 1837-2057 | 7.91-8.26 | 2.13-4.41 | 247-293 | 0-2.3 |
| 10 | 12.2-17.4 | 1981-2115 | 7.89-8.05 | 1.60-2.73 | 201-302 | 0-7.9 |
| 20 | 10.9-13.8 | 2077-2178 | 7.72-7.96 | 0.99-1.76 | 140-229 | 0.2-19.4 |
| 30 | 10.7-13.2 | 2104-2184 | 7.71-7.96 | 0.95-1.52 | 137-207 | 2.1-22.0 |
| 40 | 10.5-13.1 | 2113-2193 | 7.69-7.87 | 0.93-1.44 | 125-201 | 3.1-23.3 |
| March-July, 2018 | | | | | | |
| Depth (m) | **Temp**  **(°C)** | **DIC**  **(μmol kg^-1^)** | **pH_T_** | **Ω_Ar_** | **DO**  **(μmol kg^-1^)** | **[NO_3_^-^+NO_2_^-^]**  **(μmol kg^-1^)** |
| 0 | 16.3-25.8 | 1853-2007 | 8.08-8.25 | 2.53-4.31 | 269-314 | 0-0.1 |
| 10 | 13.7-20.0 | 1924-2084 | 7.97-8.22 | 1.92-3.38 | 238-336 | 0-4.4 |
| 20 | 12.0-15.5 | 2059-2135 | 7.84-8.00 | 1.36-2.16 | 189-235 | 0.2-15.4 |
| 30 | 11.5-13.7 | 2088-2147 | 7.81-7.95 | 1.29-1.84 | 158-226 | 0.2-16.8 |
| 40 | 11.1-13.4 | 2095-2162 | 7.76-7.93 | 1.14-1.74 | 143-216 | 1.7-19.3 |
